# Complete mitogenome of the invasive bivalve *Rangia cuneata*

**DOI:** 10.1080/23802359.2019.1659121

**Published:** 2019-09-06

**Authors:** Romain Gastineau, Brygida Wawrzyniak-Wydrowska, Claude Lemieux, Monique Turmel

**Affiliations:** a Institute of Marine and Environmental Sciences, University of Szczecin, Szczecin, Poland;; b Département de biochimie, de microbiologie et de bio-informatique, Institut de Biologie Intégrative et des Systèmes, Université Laval, Québec, Canada

**Keywords:** Baltic Sea, invasive, bivalve, Mactroidea

## Abstract

We sequenced the complete mitogenome of a Baltic Sea specimen of the invasive bivalve *Rangia cuneata*. The mitogenome is 18,993 bp long and encodes 13 proteins, 2 rRNAs, and 22 tRNAs. A large intergenic region between ND6 and ND2 contains an open reading frame that may originate from duplication of ND2. The *R. cuneata* mitogenome is rearranged in gene order relative to previously sequenced mitogenomes of Mactroidea.

A native of the American Atlantic Coast, the bivalve *Rangia cuneata* is recognized as an invasive species in Europe (Verween et al. 2006). In the early 2000s, it was discovered in the harbour of Antwerp (Belgium), where it displayed biofouling activity in the cooling water systems of an industrial plant (Verween et al. 2006). Known as a brackish-water species (Parker 1966; Swingle and Bland 1974), *R. cuneata* also settled in the Baltic Sea, probably because the low salinity of these waters favoured its spread (Rudinskaya and Gusev 2012; Warzocha and Drgas 2013; Warzocha et al. 2016; Voroshilova et al. 2018; Solovjova et al. 2019). A specimen of *R. cuneata* was collected in August 2018 from the Świna River mouth on the Baltic Sea (53°51′30.28″N 14°17′11.98″E) and registered in our field collection as KA0818-2018-RC-1; after morphological identification, a portion of this bivalve was used for DNA extraction and the remaining part was kept frozen at the University of Szczecin (Poland). Sequencing was performed on a BGISEQ-500 platform by the Beijing Genomics Institute. A total of 60 million paired-end reads of 100 bp were assembled using SPAdes 3.12.0 (Bankevich et al. 2012) and completeness of the mitogenome sequence was verified using the Consed package (Gordon et al. 1998). Genes were identified using MITOS (Bernt et al. 2013).

At 18,993 bp, the *R. cuneata* mitogenome (GenBank accession number MK878617) is the longest Mactroidea mitogenome yet sequenced. It contains 13 protein-coding genes, 2 rRNA genes, and 22 tRNA genes, all encoded on the same DNA strand. The additional length of the *R. cuneata* mitogenome relative to its Mactroidea relatives mostly lies within a 1700-bp region between ND6 and ND2 that includes 2 tRNA genes and an open reading frame coding for a hypothetical protein of 369 amino acids (orf369). Blastp analyses revealed that the latter protein displays weak sequence similarity with the ND2 proteins of two Mactroidea taxa (*Lutraria rhynchaena* and *Mactra chinensis*), raising the possibility that orf369 originated from *ND2* duplication and subsequent sequence divergence. It is well known that expansion of intergenic regions in Molluscan mitogenomes often results from events of pseudogenization (Breton et al. 2009) and that *ND2* pseudogenes are present in bivalves (Wu et al. 2010, 2012; Gastineau et al. 2018). The *R. cuneata* mitogenome is rearranged in gene order relative to previously sequenced Mactroidea mitogenomes.

A phylogenetic analysis of the protein-coding genes encoded in *R. cuneata* and other Mactroidea mitogenomes (with the exception of *atp8,* which was not available for some taxa) was carried out using RAxML version 8 (Stamatakis 2014) and the GTR + I + G model (Figure 1). *R. cuneata* strongly clustered with *Pseudocardium sachalinense* (MG431821), a clam from the Hokkaido area, also known as *Spisula sachalinense*.

**Figure 1.**
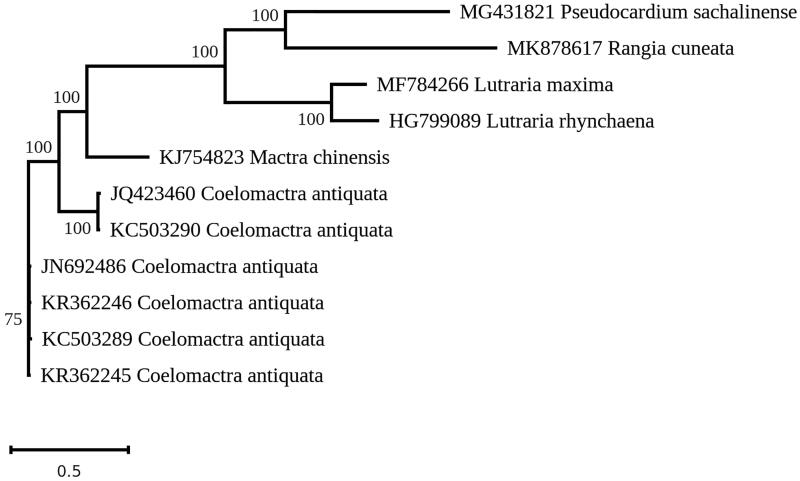
Maximum-likelihood tree obtained from concatenated mitochondrial protein-coding genes from *Rangia cuneata* and other Mactroidea, using the GTR + I + G model and after 1000 bootstrap replications.
